# Acquired CARD11 Mutation Promotes BCR Independence in Diffuse Large B Cell Lymphoma

**DOI:** 10.1200/PO.20.00360

**Published:** 2021-01-12

**Authors:** Rebecca Caeser, Ieuan Walker, Jie Gao, Nimish Shah, Livia Rasso-Barnett, Shubha Anand, Jose-Ezequiel Martin, Daniel J. Hodson

**Affiliations:** ^1^ Department of Haematology, University of Cambridge, Cambridge, United Kingdom; ^2^ Wellcome-MRC Cambridge Stem Cell Institute, Cambridge, United Kingdom; ^3^ Department of Haematology, Cambridge University Hospitals, Cambridge, United Kingdom; ^4^ Department of Haematology, Norfolk and Norwich University Hospital, Norwich, United Kingdom; ^5^ Haematopathology and Oncology Diagnostic Service, Cambridge University Hospitals, Cambridge, United Kingdom; ^6^ Cancer Molecular Diagnostic Laboratory, Department of Oncology, University of Cambridge, Cambridge, United Kingdom

Diffuse large B cell lymphoma (DLBCL) is an aggressive non-Hodgkin lymphoma that is molecularly and clinically heterogeneous. Gene expression studies have revealed how DLBCL can be divided into germinal center and activated B cell (ABC) subtypes. The ABC subtype is associated with constitutive activation of the NF-κB pathway, commonly as a consequence of genetic activation of the B cell receptor (BCR) pathway.^1^ Components of the BCR pathway that are activated by mutation include CD79B, MYD88, and CARD11. Chronic stimulation of the BCR in ABC DLBCL may also result from engagement of the BCR by self-antigens in the tumor microenvironment. These preclinical observations suggest a role for the targeted inhibitors of the BCR pathway in the treatment of DLBCL.^1^

DLBCL is most commonly present as malignant infiltration of lymph nodes. However, extranodal disease is seen in one third of cases. Some forms of DLBCL have an apparent tissue–specific restriction that allows them to be defined as specific disease subtypes. These special site forms of DLBCL include primary CNS lymphoma, primary testicular lymphoma, and primary cutaneous DLBCL (PCDLBCL). For reasons that remain mysterious, PCDLBCL most commonly involves the lower leg and PCDLBCL-leg type (PCDLBCL-LT) is recognized as a specific entity in the WHO classification of lymphoma.^2^ It predominantly affects older patients and is associated with poor response to conventional therapy.^3^ The majority of PCDLBCL-LTs exhibit an ABC DLBCL gene expression profile and are strongly enriched for genetic activation of the BCR pathway, suggesting a pathogenesis shared with nodal ABC DLBCL.^4,5^

Drugs that target the BCR pathway include inhibitors of the downstream kinases BTK, SYK, and P110delta. Inhibitors of BTK have led to dramatic responses in patients with chronic lymphocytic leukemia (CLL) and mantle cell lymphoma. The use of BTK inhibitors is now incorporated into standard therapy for these conditions, and the genetic basis of acquired resistance in CLL, dominated by acquired mutation of *BTK* or *PLCG2*, has been well-studied.^6,7^ By contrast, the role of BCR inhibition in DLBCL remains less clear and little is known about the genetics of acquired resistance.

A 75-year-old male presented with rapidly enlarging cutaneous nodules. These initially involved the feet and legs, but subsequently emerged on the arms and abdomen. A computed tomography scan showed no lymph node enlargement. Biopsy revealed sheets of large lymphoid cells expressing PAX5, CD20, BCL2, MUM1, and weak BCL6. CD10 staining was negative. The proliferation fraction was 90%. A diagnosis of PCDLBCL was made. Because of comorbidity, he was initially treated with rituximab, gemcitabine, cyclophosphamide, vincristine, and prednisolone with partial response. At relapse two years later, he was retreated with rituximab and gemcitabine, and subsequently oral etoposide with minimal response to either therapy. He was referred to our tertiary lymphoma service for further management. Clinical examination revealed cutaneous nodules up to 25 mm in diameter affecting the feet, legs, arms, and abdomen. A repeat biopsy showed features identical to his original diagnostic biopsy (Fig 1A), and computed tomography scan confirmed exclusively cutaneous disease. He was commenced on oral therapy with BTK and SYK inhibitors. By day 28, he had a near-complete resolution of his cutaneous lesions, which further improved by 6 months (Fig 1B). With ongoing therapy, his remission lasted for 13 months before the recurrence of rapidly growing cutaneous nodules and lymph node involvement. A repeat biopsy confirmed recurrent DLBCL with immunophenotype identical to his diagnostic biopsy. The patient was managed with palliative radiotherapy.

**FIG 1.**
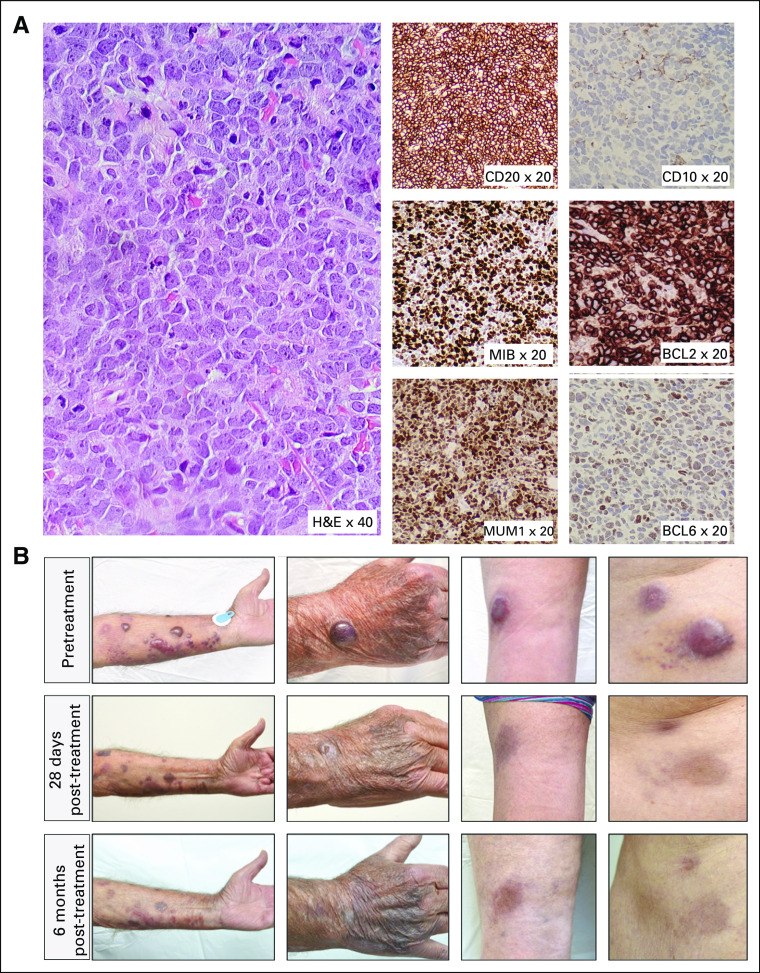
(A) Histology and immunohistochemistry from tumor biopsy showing a diffuse infiltration by large atypical cells with predominantly centroblast-like morphology expressing CD20, MUM1, BCL2, weak BCL6, no CD10, and MIB1 proliferation fraction 90%. (B) Images of selected lesions on the forearm, hand, popliteal fossa, and abdomen taken prior to therapy (top row), at 28 days (middle row), and at 6 months (bottom row) of B cell receptor–targeted therapy. H&E, hematoxylin and eosin.

The rapid clinical response and prolonged remission, followed by later re-emergence of tumor, suggested the acquisition of new genetic alterations driving resistance to BCR inhibition. We therefore performed whole-exome sequencing using DNA extracted from biopsies at initial diagnosis, immediately prior to BCR inhibition, and at relapse. The patient provided written informed consent for genetic analysis and publication of clinical photographs. This study was approved by the East of England Cambridge South Research Ethics Committee (approval reference number 07/MRE05/44).

Full variant and copy number data are presented in the Data Supplement. Notable genetic alterations detected prior to BCR inhibition included amplification of Chr18q21 (including *BCL2*, *MALT1*, and *TCF4*), amplification of Chr13q31 (including *miR17-92*), and homozygous deletions of *GNA13 and RB1* (Fig 2A and Appendix Fig A1). We observed gains of *CD79A* and *SPIB* and single copy loss of *PRDM1* and *TNFAIP3* (A20). We did not identify mutation of *MYD88* or *CD79B* (Fig 2A and Appendix Fig A2). Importantly, many of the identified genetic alterations are predicted to enhance activity of the BCR and NF-κB pathways^1^ (Fig 2B). These data suggest that this patient's tumor matched the biology of ABC DLBCL and relied on chronic active BCR signaling to maintain NF-κB activity.

**FIG 2.**
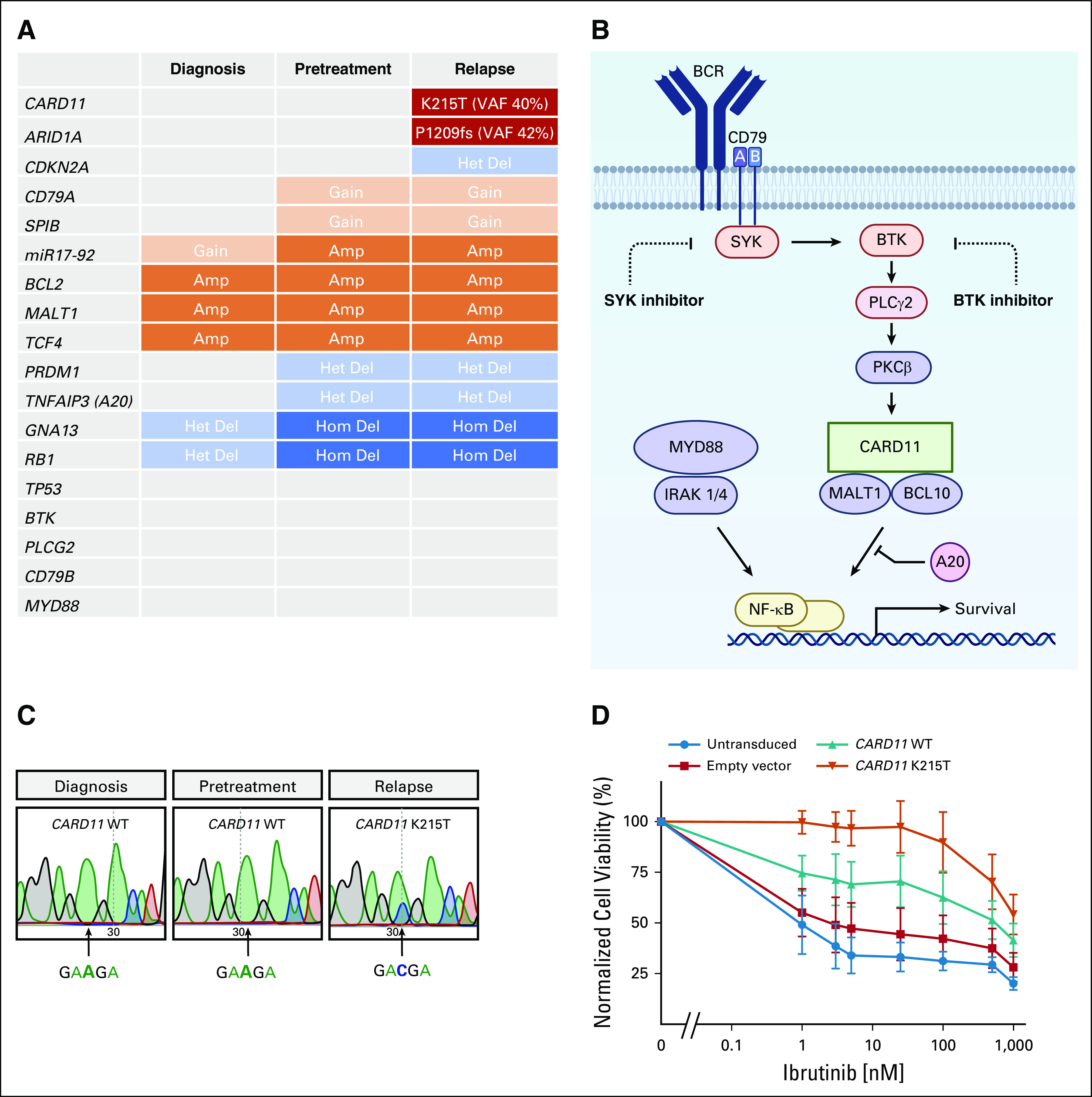
(A) Summary of whole-exome sequencing of samples taken from three different timepoints; diagnosis, prior to BCR-targeted therapy, and at the time of relapse. Mutations and copy number changes are indicated within each cell. Gray indicates no mutation or copy number change identified. (B) A simplified schematic showing the critical signaling components of the BCR pathway that converge onto activation of canonical NF-κB. Components targeted pharmacologically in this patient are indicated. Created with BioRender.com. (C) Sanger sequencing from samples at the indicated timepoints confirms the acquisition of an A to C mutation in CARD11, which results in the activating, coiled-coil domain K215T mutation. (D) Ibrutinib sensitivity assay in the ABC DLBCL line U2932 transduced with either empty vector or CARD11 WT or K215T. Data show mean and SEM of five independent experiments. BCR, B cell receptor; VAF, variant allete frequency.

We then screened all genetic alterations acquired during BCR inhibition to identify those that might drive resistance. We identified 34 protein-altering mutations that arose during exposure to BCR inhibition, including the known DLBCL driver genes—*ARID1A* and *CARD11* (Figs 2A and 2B and Data Supplement). Copy number analysis showed a new single copy loss of the *CDKN2A* locus (Data Supplement). We manually examined the loci of *PLCG2* and *BTK*, mutations that are commonly acquired in BTKi-resistant CLL, and confirmed both genes to be wild type at all timepoints. Alteration of *TP53* was not detected. The majority of altered genes lacked any known association with BCR signaling. *CDKN2A* loss is common in ABC DLBCL; however, we considered it unlikely that single copy loss could mediate resistance to BCR inhibition. In contrast, CARD11 is a critical scaffold protein that, together with BCL10 and MALT1, functions downstream of SYK and BTK in the BCR pathway to activate canonical NF-κB signaling.^1^ Activating mutation in the coiled-coil domain of CARD11 was previously proposed as a mechanism of resistance to BCR inhibition in mantle cell lymphoma^8^ and primary resistance in DLBCL.^9^ The acquired K215T mutation in our patient is located in the coiled-coil domain and has been previously shown to activate NF-κB.^10^ We confirmed the near-clonal presence of the *CARD11* K215T mutation at relapse (variable allele frequency 40%) but zero mutant reads at this position in either of the two pretreatment biopsies (read depth, 180 and 210, Appendix Fig A3). Sanger sequencing verified the acquisition of the activating *CARD11* K215T mutation in the relapse sample but not at diagnosis or in the pretreatment sample (Fig 2C). To establish the ability of this mutation to mediate resistance, we expressed *CARD11* WT or K215T in the ABC DLBCL line U2932 and treated cells with the BTK inhibitor ibrutinib. *CARD11* K215T induced drug resistance, with IC50 increased by three orders of magnitude (Fig 2D). Taken together, these data strongly suggest that *CARD11* mutation was the genetic driver of the acquired resistance in this case.

The limited information about acquired resistance to BCR inhibition in DLBCL suggests potential differences compared with that of CLL. The latter is predominantly associated with mutation of *BTK* and *PLCG2*.^6,7^ *CARD11* mutation is identified in 10%-15% of DLBCL at presentation^11^ but is rarely identified (< 1%) in CLL.^12^ This suggests that the BCR signal in CLL and DLBCL may be qualitatively different and therefore that genetic mechanisms of resistance to BCR inhibition may also differ. However, establishing the genetic basis of resistance to BCR inhibition in DLBCL presents specific challenges that contrast with the situation in CLL. First, patients with DLBCL receiving BCR inhibitors are typically treated simultaneously with multiagent immunochemotherapy regimens, making it hard to establish which mutations provide resistance to which agent, or indeed if sensitivity to the BCR inhibitor ever existed in the first place. Second, relapsing DLBCL frequently presents as a rapidly progressive disease that may preclude the opportunity for a repeat biopsy in patients who are often treated palliatively. These factors make analysis of rare exceptional responders especially important for DLBCL. The genetics of our case recapitulate the biology of ABC DLBCL, and importantly, we observed clear initial sensitivity to therapy targeted exclusively to the BCR pathway. Combined with the biopsy-accessible cutaneous location of disease, this provided a rare opportunity to study genetic mechanisms of resistance to BCR inhibition in ABC DLBCL.

Indeed, we have found only one other case describing the genetic basis of acquired resistance to BCR inhibition in DLBCL. This also involved a case of PCDLBCL-LT and also reported acquisition of *CARD11* mutation (interesting also K215 mutant) in a patient who relapsed following an initial response to a BTK inhibitor.^13^ However, the concurrent finding of *NFKBIE* mutation and *IgH-IRF8* translocation reported in that study might also have contributed to resistance, leaving the role of the *CARD11* mutation uncertain. Our findings of acquired activating *CARD11* mutation, the absence of any likely alternative genetic explanation, and the demonstrated ability of the K215T mutation to affect BTKi resistance in vitro strongly suggest that *CARD11* mutation is the dominant driver of BCR independence in our case.

As we move toward the introduction of precision medicine in DLBCL and the real-time monitoring of clonal evolution, it will become increasingly important to understand genetic mechanisms of drug resistance. Our study uses the unique features of this case of cutaneous DLBCL to highlight the importance of *CARD11* mutation as a driver of acquired resistance to BCR inhibition in DLBCL. We speculate that such cases might be targeted successfully in the future by inhibition of downstream targets such as MALT1 or NF-κB.^14,15^

## Figures and Tables

**FIG A1.**
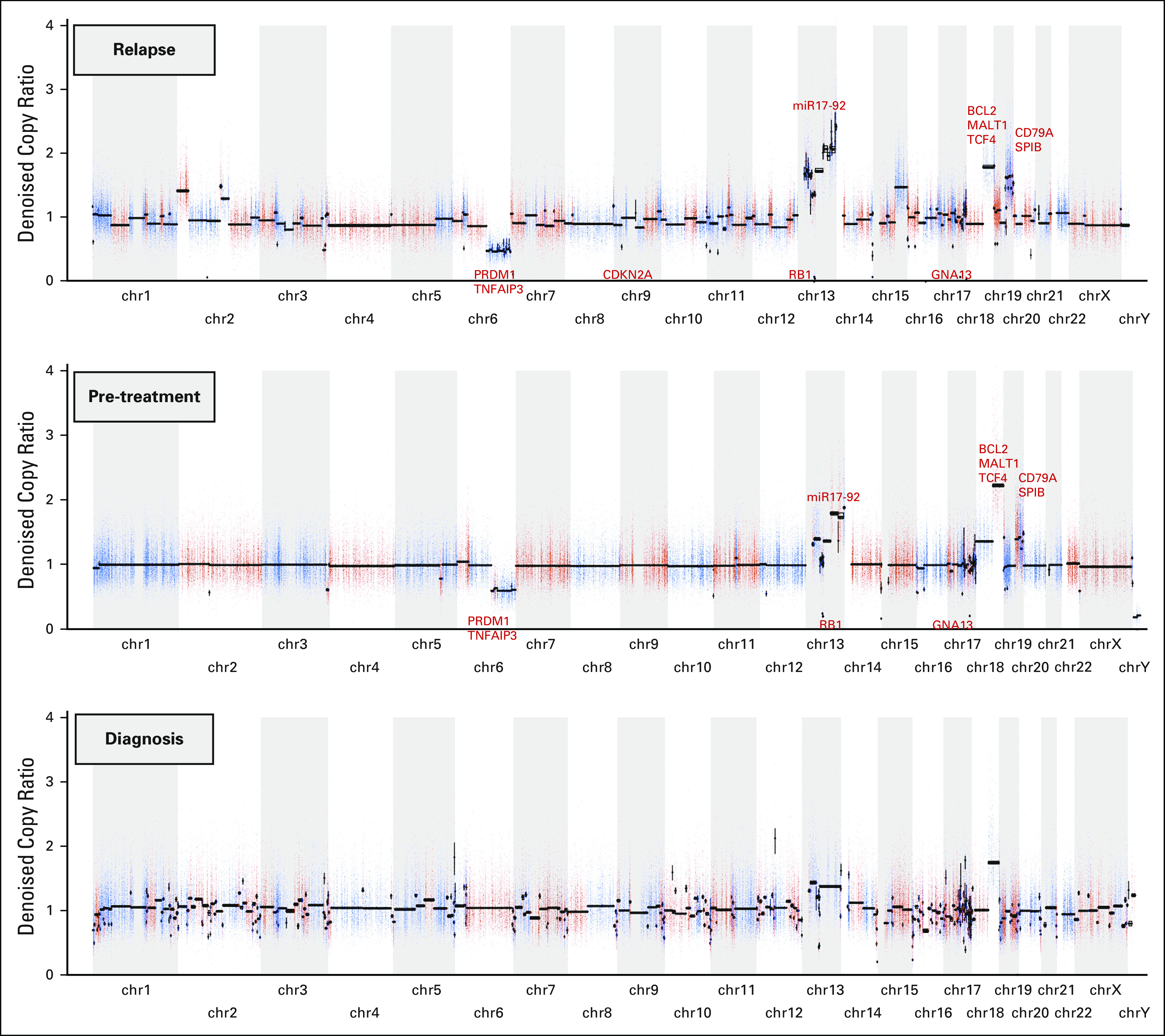
Copy number change detected at the indicated timepoints. The position of known lymphoma driver genes is indicated.

**FIG A2.**
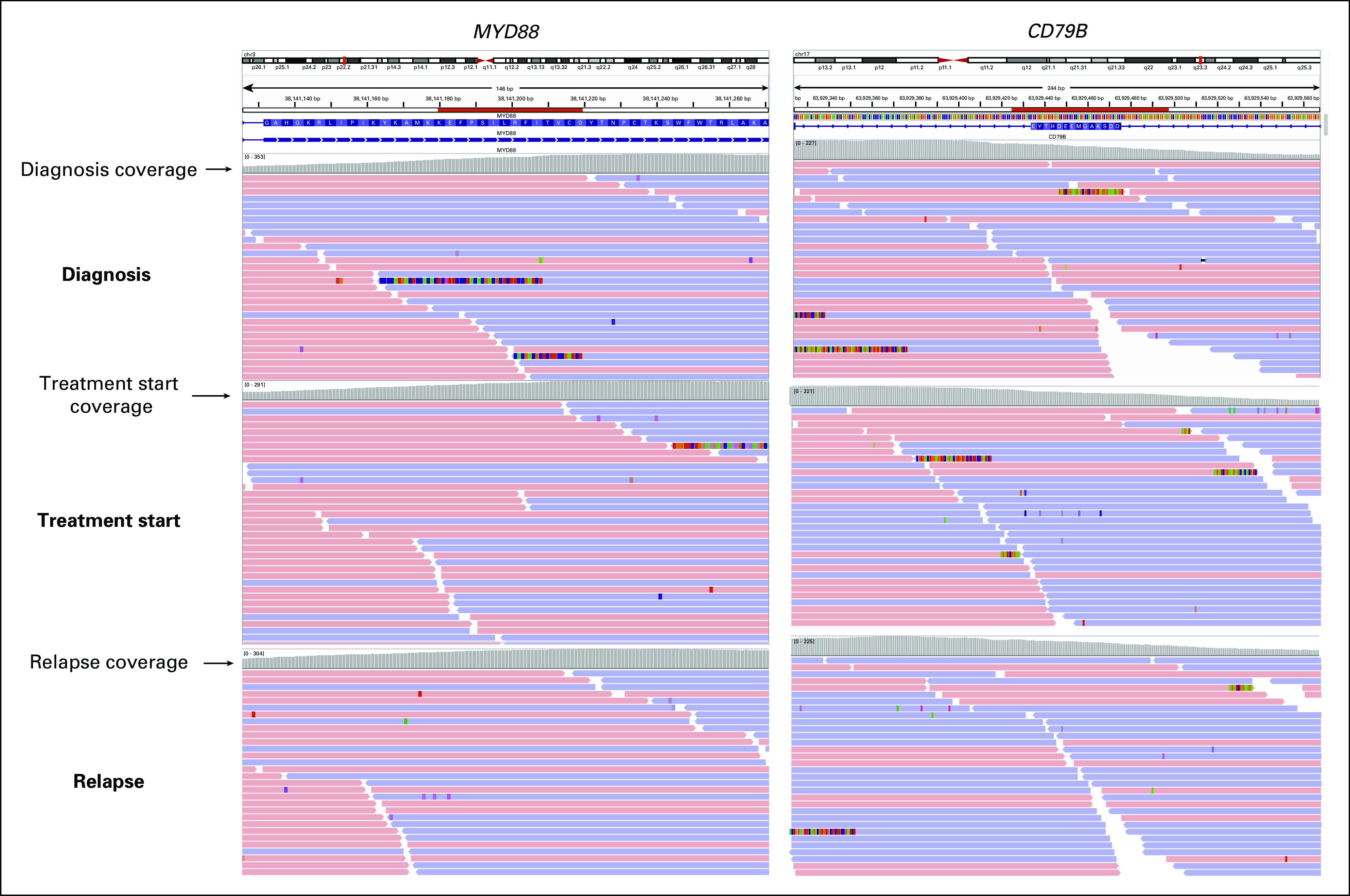
Summary of exome sequencing reads mapping to mutation hotspot regions of *MYD88* and *CD79B*, confirming that both genes are wild type at these genomic locations at each timepoint.

**FIG A3.**
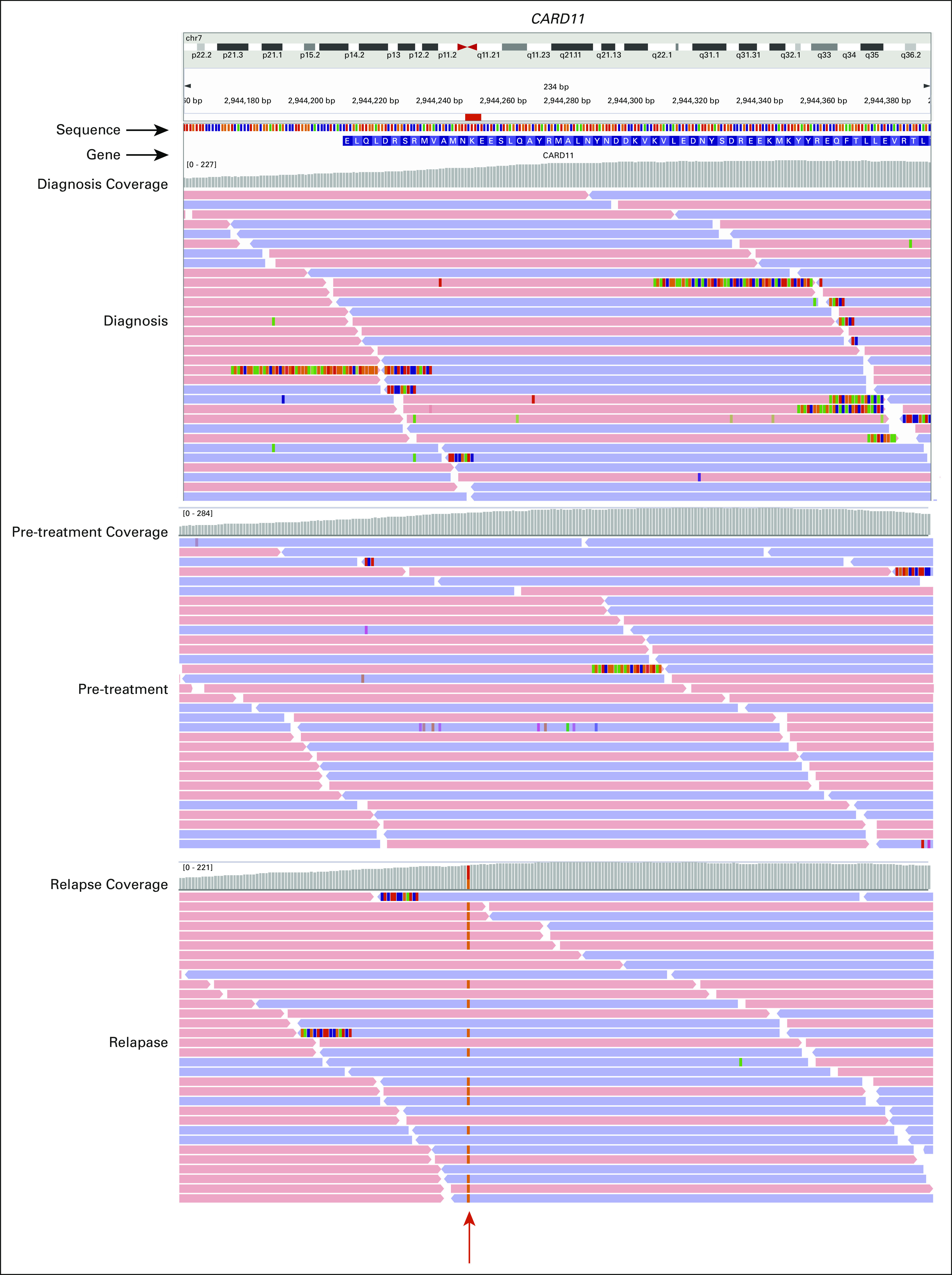
Summary of exome sequencing reads mapping to the region of the *CARD11* locus containing detected variant at the indicated timepoints, showing acquisition of the *CARD11*_K215T mutation (red arrow) at relapse.
